# Supplementary material for: Multi-indicator sustainability assessment of global food systems
Source: Nat Commun. 2018 Feb 27;9:848. doi: 10.1038/s41467-018-03308-7 (PMC5829192; doi:10.1038/s41467-018-03308-7)
Supplement: Supplementary file 2 — Description of Additional Supplementary Files [file 41467_2018_3308_MOESM2_ESM.pdf]

Description of Additional Supplementary Files

File Name: Supplementary Data 1
Description: Normalized scores (0-100) of seven food system metrics and 25 indicators for 156 countries in the year 2011. Metric score is arithmetic average of its underlying indicator scores.

File Name: Supplementary Data 2
Description: Normalized scores (0-100) of seven food system metrics and 25 indicators for World Bank regions.

File Name: Supplementary Data 3
Description: Population share of Adequate Nutrients (PAN) values for 17 nutrients considered in this study and calculated using EAR-CP approach under current (2011) diets. PAN values under three alternative dietary scenarios (HGD, VGT and VGN) are also shown.

File Name: Supplementary Data 4
Description: All food items contributing to >10% of the total national carbon and water footprint for each 156 countries.

File Name: Supplementary Data 5
Description: Spearman rank correlation coefficient between all 25 indicators, seven metrics and GDP $capita^{-1}$ of 156 countries.

File Name: Supplementary Data 6
Description: Changes in national nutrition balance score (NBS), disqualifying nutrient score (DNS), population with adequate nutrition (PAN), carbon footprint (gCO2eq. $capita^{-1}$ day^{-1}) and water footprints (liters $capita^{-1}$ day^{-1}) under three dietary change scenarios (HGD, VGT and VGN). REF corresponds to current diets. See Table 2 for region-aggregated results.

File Name: Supplementary Data 7
Description: List of all 94 food items for which daily food supply data was downloaded from FAO's food balance sheet (FBS). The individual products comprising each of the 94 items are also shown (see methods for details).
